# Has the distribution of smoking across young adult transition milestones changed over the past 20 years? Evidence from the 1970 British Cohort Study (1996) and Next Steps (2015–16)

**DOI:** 10.1016/j.ssmph.2021.100941

**Published:** 2021-10-09

**Authors:** T. Gagné, I. Schoon, A. Sacker

**Affiliations:** a Department of Epidemiology and Public Health, University College London, UK; b International Centre for Lifecourse Studies in Society and Health, UK; c Institute of Education, University College London, UK

**Keywords:** Great Britain, Smoking, Young adults, Transition to adulthood, 1970 British cohort, Next Steps cohort

## Abstract

**Introduction:**

Transitions into work and family life during young adulthood exacerbate differences in the progression of smoking over the life-course. Few have considered how changes in smoking and the transition to adulthood in the past two decades have influenced these relationships over time.

**Methods:**

We compared the distribution of smoking at ages 25–26 across transition milestones among 3764 men and 4568 women in the 1970 British Cohort study (1996) and 3426 men and 4281 women in the Next Steps study (2015–16). We regressed occasional and daily smoking status on educational attainment, economic activity, living arrangements, relationship status, and parenthood, adjusting for family background, socio-demographics, and smoking history.

**Results:**

There were few differences in associations between the 1996 and 2015-16 samples. Young men and women were less likely to smoke if they had higher education, were homeowners, and cohabited with a partner. Women were less likely to smoke occasionally if they were full-time students, and men were less likely to smoke daily if they were employed full-time and not living with children. However, comparing associations in 2015–16 to 1996: 1) in men, higher education had a weaker negative association and living with a partner had a stronger negative association with daily smoking; 2) in women, independently renting had a weaker positive association with daily smoking.

**Conclusions:**

Despite considerable changes in smoking and the transition to adulthood over the past two decades, the distribution of smoking at ages 25–26 across transition milestones has been relatively stable during this time period in Great Britain.

## Highlights

• Smoking at ages 25–26 was associated with economic activity, living arrangements, relationship status, and parenthood in 1996 and 2015–16.
• Differences in smoking across transition milestones differed by gender.
• Differences in smoking did not substantially change between 1996 and 2015–16.

## 1 Introduction

Cigarette smoking represents a major determinant of morbidity and premature mortality, and is one of the core mechanisms driving health inequalities (Petrovic et al., 2018). Smoking cessation in young adulthood may fully counter the effect of smoking on later-life health (Taylor et al., 2002; Kenfield et al., 2008). Tobacco control efforts in this age group, however, face three challenges: 1) young adults have a higher smoking prevalence (in the United Kingdom (UK), 16% among those aged 18–24 and 19% among those aged 25–34 compared to 14% in the adult population in 2019) and lower cessation rate than older age groups (Brown & West, 2017; Public Health England, 2020), 2) they experienced fewer gains from interventions designed to reduce initiation compared to younger age groups over the past fifteen years (Cantrell et al., 2018; Gagné & Veenstra, 2017), 3) tobacco control historically favoured interventions for adolescents, leaving young adults with fewer age-sensitive interventions to prevent initiation and support cessation (Fanshawe et al., 2017; Villanti et al., 2020).

A massive literature has portrayed the social distribution of smoking at different life periods including young adulthood (Gagné et al., 2019a; Schaap & Kunst, 2009). Proximal mechanisms explaining differences across social groups include: nicotine dependence, lack of support in keeping with the higher acceptability of smoking in their social network and environment, focus on current issues over later-life health, stress and boredom, and reduced capacity to follow-up on cessation methods (Hiscock et al., 2012a). Differences persist today: in 2018, English young adults aged 18–34 were 139% more likely to smoke if they were employed in routine-manual occupations (*vs.* managerial and professional occupations) and 78% more likely to smoke if they resided in a neighbourhood in the bottom decile of deprivation (*vs.* the top decile). (Public Health England, 2020).

Whereas the social distribution of health behaviours is often assessed using socioeconomic indicators (Galobardes et al., 2006), researchers have promoted a life-course perspective to better understand behavioural changes during the transition to adulthood, considering variations across the transition milestones encompassing the move out of education into work and family formation during this life period. Studies across Canada, the United States, and the UK have shown that – beyond social background and educational attainment – reaching the social roles associated with entering a full-time job, leaving the parental home, starting a committed relationship, and having children each contribute to the likelihood of smoking (Gagné et al., 2020; Graham et al., 2006; Green et al., 2017; Pampel et al., 2014; Staff et al., 2010; Wickrama & Baltimore, 2010). Considering them may help better understand the conditions – i.e., resources, networks, environments, and norms – that leave less privileged young adults with a lower capacity to avoid smoking (Link & Phelan, 2009; Poland et al., 2006). Taking into account the challenges faced by young adults in more recent years may also help support the design of interventions targeting young adult smokers across the socioeconomic spectrum (Greaves et al., 2019; Kock et al., 2019; Villanti et al., 2020).

An issue relatively unexplored in this field concerns the variability of the associations between transition milestones and smoking in young adulthood over time, and the potential impact that social change may have had on these associations. Despite increases in higher education participation, poorer economic conditions – e.g., decrease in real income and rise in student debt, precarious forms of work, and housing prices – have led this age group to become increasingly worse off over the past decades (Henderson, 2019; Schoon & Bynner, 2019; Sironi, 2018). Following this increased insecurity, transitions out of the parental home into independent living, cohabitation with a partner, and parenthood have been delayed into later decades of life (Office for National Statistics, 2019a). These changes have led more to delay role transitions such as full-time employment and cohabitation with a partner which are positively associated with smoking cessation (Henkel, 2011; Homish & Leonard, 2005; McDermott et al., 2006). Increased uncertainty and precarity, however, may also have made these transitions more stressful, potentially leading more to maintain smoking as a coping mechanism (Green et al., 2017).

Some have already explored how the social distribution of smoking during young adulthood may have changed over time. Studies used age-period-cohort analyses in the United States, France, and Germany and found that educational differences in smoking during the transition to adulthood had increased across cohorts born between the 1940s and 1980s in the United States and France, but not so much in Germany (Bricard et al., 2016; Pampel et al., 2015, 2017). A British study instead found that smoking at ages 23–26 had been more strongly associated with education in those born in 1958 compared with those born in 1970, corroborating the potential for differences across countries (Green et al., 2017). To our knowledge, no study has examined how changes in young adult milestones across education, employment, family, and housing relate to changes in their association with smoking in more recent years, particularly in the UK context.

### 1.1 Objectives

Studies on changes in the social distribution in smoking over time have been predominantly informed in the adult population using socioeconomic indicators such as education, occupation, and income. These indicators do not capture the full extent of the progression of smoking during young adulthood. Beyond differences attributable to social background and smoking initiation in early life, the goal of this paper is to compare differences in smoking across transition milestones – i.e., educational attainment, economic activity, independent living, partnership status, and parenthood – across two British samples surveyed at ages 25–26 twenty years apart, in 1996 and 2015–16. While these findings cannot directly point to policy solutions, they may help better understand which high-risk groups should be prioritised in prevention measures and communication strategies designed for this age group. The age of 25–26 is particularly relevant for smoking as nearly 100% of smokers are expected to have initiated smoking by this point (U.S. Department of Health and Human Services, 2012). Given changes in young adult transition milestones since the 1990s – i.e., rise in higher education participation; declines in viable forms of employment, marriage, and home ownership; delays in independent living and parenthood, we expect that, at ages 25–26: 1) higher education could be associated to a smaller degree with smoking, 2) social role transitions that have become more difficult to negotiate, such as the move in work (e.g., full-time employment), family (e.g., partnership), and housing (e.g., home ownership), will be associated to a larger degree with smoking.

## 2 Methods

### 2.1 Data

We use two datasets. The 1970 British Cohort Study recruited 17,196 individuals born during a single week in April 1970 across the UK and followed them again at ages 5, 10, 16, 26, 30, 34, 38, 42, and 46–48 (University of London and Ins, 2016). At age 26 (Apr 1996/Sep 1996), 3764 men and 3568 women participated, representing a 68% response rate among eligible cases. The Next Steps study, also known as the Longitudinal Study of Young People in England (LSYPE), recruited 15,770 young people aged 13–14 in 2004 (i.e., born in 1989–90), inviting them to participate each year until ages 19–20, and another time at ages 25–26 (University College London, 2020). At ages 25–26 (Aug 2015/Sep 2016), 3426 men and 4281 women participated, representing a 51% response rate among eligible cases.

### 2.2 Measures

Smoking status was measured with the same item in the 1970 and Next Steps cohorts, asking “which of the following describes your smoking habit?”, with four response options including “I've never smoked”, “I used to smoke cigarettes but don't at all now”, “I now smoke cigarettes occasionally but not every day”, and “I smoke every day”. We combined never- and former-smokers and coded participants into: 1) non-smokers, 2) occasional smokers, and 3) daily smokers. We examined occasional and daily smoking separately as: 1) smoking frequency has decreased over time, 2) occasional smoking is more prevalent among young adults compared to older age groups, 3) the prevention of the progression to daily smoking is promoted in this age group, 4) occasional and daily smoking are expected to be associated with different social characteristics (Office for National Statistics, 2019b; Villanti et al., 2019).

Transition milestones assessed at ages 25–26 included educational attainment, economic activity, independent living, partnership status, and parenthood. *Educational attainment* was measured with the National Vocational Qualifications (NVQ) scheme. NVQ1 and NVQ2 refer to: 1) in the 1970 British Cohort, the Certificate of Secondary Education and O-levels (ended in late 1980s) and 2) in the Next Steps cohort, lower and higher grades in the General Certificate of Secondary Education examinations. NVQ3, NVQ4, and NVQ5 represent pre-university training (A-levels), sub-degree programs (further education), and university degrees (higher education), respectively. *Economic activity* was measured using a derived variable coded into: 1) full-time employed, 2) part-time employed, 3) unemployed, 4) full-time student, 5) other (e.g., homemaker, long-term disability). *Independent living* was coded based on information on cohabitation and housing tenure into: 1) living with parents rent-free; 2) without parents, owning; 3) without parents, renting; 4) other (e.g., squatting, boarding, rent-free elsewhere, paying rent to parents). Response options precluded us from separating those who lived with parents and had an informal arrangement where they paid board to them from those in “other” arrangements. *Partnership status* was coded based on information on cohabitation and marital status into: 1) single (not cohabiting), 2) cohabiting with a partner, 3) married, and 4) divorced, separated, or widowed. Finally, *parenthood* was recoded based on information on cohabitation into: 1) no children, 2) one child, or 3) two or more children. We used “living with children” as a proxy of parenthood that includes non-biological children.

To address confounding, we used comparable measures of social background and health in adolescence at ages 0, 10, and 16 in the 1970 cohort and ages 13–14 in the Next Steps cohort. *Parents' education* was measured based on the highest age among parents when they left education into: ages 1) ≤16, 2) 17–18, 3) ≥19. We chose these categories based on the ages at which mandatory schooling ends (increased from age 15 to 16 in 1971–72) and higher education usually starts. *Parents' home ownership* was measured into: 1) Owner, 2) Renter. *Mother's age at birth* was coded into ages 1) <20, 2) 20–24, 3) 25–29, 4) ≥30. To capture health in adolescence, we used similar items asked to participants' parents (in the 1970 cohort: “Does your child have any medical condition or illness, any behaviour problem or educational difficulty which you consider to be important?” and “Does it affect everyday life at home or at school?”; in the Next Steps cohort: “Does (your child) have any long-standing illness, disability or infirmity?”, “Do these problems make it harder for (your child) to go to school or college regularly?”, and “Do these problems affect (your child)'s ability to do his/her school work?”). We recoded *adolescent health* into: 1) no problems, 2) problems, no limitations, 3) problems, with limitations. We considered as additional covariates whether participants *lived in England* at ages 25–26 (Yes/No) and *ethnic group* (1970: 1) White, 2) Non-White; Next Steps: 1) White, 2) Mixed or Other, 3) South Asian, 4) Black).

To capture participants’ *smoking history* and minimize potential selection bias (i.e., early uptake of smoking predicting milestones at ages 25–26), we used a measure of the age at which participants started smoking daily for one continuous year, coding them into: 1) started by age 16, 2) did not start by age 16 (coinciding with the end of mandatory schooling). We did not further distinguish ages after age 16 as smoking could have been initiated after reaching milestones, leading to over-adjustment (e.g., leaving full-time education at age 16 and then starting smoking). This measure was asked at ages 25–26 in the Next Steps cohort and at the ages 42 and 46–48 waves in the 1970 cohort. We further reduced the number of missing cases on this variable in the 1970 cohort using data on the age at which participants last smoked at the ages 16, 30, 34, and 38 waves.

### 2.3 Statistical analyses

For the main analyses, we used multinomial logistic models, regressing smoking status at ages 25–26 on the five transition variables, adjusting for social background, adolescent health, country, ethnic group, and smoking history. Given that the prevalence of smoking and transition milestones, and the magnitude of their association, differ between men and women, we produced analyses for each cohort (1970/Next Steps) and sex (Men/Women) separately. In each case, we report the relative risk ratios of smoking across the categories of independent variables in partially-adjusted (transitions separately) and fully-adjusted models (transitions together). Relative risk ratios can be interpreted as a special form of odds ratios based on a reference category (i.e., not smoking) when regressing a nominal outcome, whereas odds ratios are computed from dichotomous outcomes. We note that differences between these partially- and fully-adjusted models may include both confounding and mediation effects, particularly for educational attainment as it is likely to end before other transitions start. We used a seemingly unrelated estimation (SUEST) procedure to test differences in relative risk ratios across cohorts (Supplementary Table 2) (Weesie, 1999). SUEST is equivalent to a model pooling the two samples where we add interaction terms between cohort and each independent variable (Mize et al., 2019). We chose SUEST over a pooled-sample approach to test differences because the associations of covariates with transition milestones and smoking may vary across cohorts. To examine differences on the absolute scale, we also estimated differences in marginal probabilities of occasional or daily smoking across the categories of independent variables based on the fully-adjusted models (Supplementary Tables 3–4). As a sensitivity analysis, we reproduced the main analyses using a four-category version of the outcome (never, former, occasional, and daily smoker), no longer controlling for the “smoking history by age 16” covariate (Supplementary Tables 5–6). Whereas this analysis corroborates the main findings, we discuss some key differences in the next section.

Attrition between the time participants first enter the cohort and the last wave at ages 25–26 is a potential source of bias. Differential attrition is relatively common in longitudinal cohort datasets and should be addressed (Mostafa & Wiggins, 2015). Detailed discussion of attrition in these datasets is available in Mostafa & Wiggins (2015) and the Next Steps User Guide (2018) (Mostafa & Wiggins, 2015; Calderwood, 2018). We adjusted for attrition in the two datasets by weighting (Mostafa & Wiggins, 2015). For the Next Steps cohort, we used the weight, sampling unit, and strata variables developed by the Next Steps team to account for the complex survey design, baseline non-response, and attrition by ages 25–26. For the 1970 cohort, we created an inverse-probability weight for attrition at age 26, matching predictors used in the Next Steps dataset using data on the parents' education, employment status, and social class; the mother's marital status; and the participant's sex, weight, and country of residence at birth. We note that, compared with the Health Survey for England (HSE), the weighted prevalence of smoking in the 1970 cohort matched the 1996 HSE estimate but the weighted estimate in the Next Steps cohort was higher compared to the 2015-16 HSE estimates (Smoking. Health Survey fo, 2020). To account for missingness, we used multiple imputation by chained equations and performed analyses in 20 imputed datasets (Royston & White, 2011). We compare the distribution of study variables in available and imputed samples in Supplementary Table 1. Analyses were produced using Stata 16 (Statacorp, 2019). Stata .do files were uploaded on the Open Science Framework platform to facilitate the reproduction of results (OSF, 2021).

## 3 Results

### 3.1 Sample characteristics

Table 1 presents the distribution of smoking status, transition milestones, and covariates at ages 25–26 by sex in each cohort, based on estimates produced after weighting and multiple imputation. In the 1970 cohort, 11% of men and 10% of women were occasional smokers, and 29% of men and 27% of women were daily smokers. In the Next Steps cohort, 13% of men and 11% of women were occasional smokers, and 21% of men and 19% of women were daily smokers. Regular smoking by age 16 was relatively similar across cohorts.

**Table 1:** Sample characteristics. 1970 British Cohort Study (1996) and Next Steps study (2015–16).

Variable	1970 BCS (1996) *N* = 8332				Next Steps (2015–16) *N* = 7707			
	Men *N* = 3764		Women *N* = 4568		Men *N* = 3426		Women *N* = 4281	
	N	WI%	N	WI%	N	WI%	N	WI%
**Smoking status**								
Non-smoker	2267	60.8	2907	63.6	2328	65.8	3180	70.1
Occasional smoker	409	10.7	441	9.6	413	12.8	405	11.0
Daily smoker	1037	28.5	1171	26.8	550	21.4	545	18.9
*Missing*	*51*	*1.4*	*49*	*1.1*	*135*	*4.2*	*151*	*3.5*
**Education (NVQ)**								
No qualifications	385	12.3	522	13.1	256	9.3	287	8.6
NVQ 1	235	7.5	368	9.1	387	17.5	349	13.3
NVQ 2	982	29.4	1333	32.2	794	26.3	884	23.9
NVQ 3	550	15.9	530	12.4	632	15.5	874	17.4
NVQ 4	1059	29.5	1229	28.0	805	19.2	1185	24.5
NVQ 5	192	5.4	235	5.3	550	12.3	695	12.3
*Missing*	*361*	*9.6*	*351*	*7.7*	*2*	*0.1*	*7*	*0.2*
**Economic activity**								
FT employed	3139	83.4	2926	63.2	2641	75.0	2727	57.8
PT employed	88	2.4	570	13.1	250	7.2	620	17.2
Unemployed	259	7.5	117	2.8	256	9.2	246	6.3
FT student	118	3.1	95	2.1	164	4.2	228	5.1
Other	126	3.5	796	18.9	92	4.4	430	13.5
*Missing*	*34*	*0.9*	*64*	*1.4*	*23*	*0.7*	*30*	*0.7*
**Independent living**								
With parents rent-free	929	25.4	787	17.4	974	26.6	973	19.7
W/out parents, owning	1385	36.2	2066	44.9	513	15.1	797	18.5
W/out parents, renting	1002	27.1	1327	30.0	1046	34.3	1589	43.6
Other	400	11.3	345	7.7	881	24.1	900	18.2
*Missing*	*48*	*1.3*	*43*	*0.9*	*12*	*0.4*	*22*	*0.5*
**Relationship status**								
Single	1857	49.9	1587	34.7	2321	64.5	2457	53.9
In couple	913	24.2	1144	25.1	786	27.4	1165	32.1
Married	888	23.9	1598	35.4	303	7.7	611	12.9
Div./Sep./Widowed	74	2.0	214	4.8	10	0.3	43	1.1
*Missing*	*32*	*0.9*	*25*	*0.5*	*6*	*0.2*	*5*	*0.1*
**Living with children**								
No children	3156	82.8	3186	68.3	3036	84.2	3207	67.3
One child	410	11.5	775	17.3	239	9.2	587	16.3
2+ children	198	5.6	607	14.3	151	6.6	487	16.4
*Missing*	*0*		*0*		*0*		*0*	
**Smoking history**								
Reg. smoking by 16	718	24.9	933	25.0	531	22.5	689	24.4
Not reg. smoking by 16	2492	75.1	3126	75.0	2756	78.5	3436	75.6
*Missing*	*554*	*14.7*	*509*	*14.3*	*139*	*4.3*	*156*	*3.6*
**Health at ages 10/13**–**14**								
No problems	2422	73.4	3114	77.6	2755	81.8	3644	86.7
Problems, no limitations	347	11.0	410	10.6	299	10.0	247	6.7
Problems, limitations	509	15.6	468	11.8	220	8.2	204	6.5
*Missing*	*486*	*12.9*	*576*	*12.6*	*152*	*4.7*	*186*	*4.3*
**Mother's age at birth**								
Less than 20	324	9.9	340	8.5	202	7.9	275	8.5
20–24	1324	35.2	1645	36.0	784	25.7	997	26.0
25–29	1210	30.7	1510	31.7	1150	35.2	1379	33.2
30 or more	880	24.2	1061	23.8	1098	31.2	1401	32.3
*Missing*	*26*	*0.7*	*12*	*0.3*	*192*	*5.9*	*229*	*5.3*
**Parents' education**								
16 or less	2708	77.0	3304	77.0	1558	46.7	2022	46.2
17–18	409	12.7	534	13.3	775	23.4	947	23.9
19 or older	317	10.2	368	9.8	1040	29.9	1235	29.8
*Missing*	*330*	*8.8*	*362*	*7.9*	*53*	*1.6*	*77*	*1.8*
**Parents' housing**								
Owner	2382	62.9	2904	63.6	2520	69.0	3058	67.8
Not owner	1228	37.1	1496	36.4	821	31.0	1087	32.2
*Missing*	*154*	*4.1*	*168*	*3.7*	*85*	2.6	*136*	*3.2*
**Country at ages 25**–**26**								
In England	3015	84.8	3717	86.5	3390	99.1	4229	99.3
Not in England	523	15.2	571	13.5	25	0.9	34	0.7
*Missing*	*226*	*6.0*	*280*	*6.1*	*11*	*0.3*	*18*	*0.4*
**Ethnic group**								
White	3481	93.5	4265	94.3	2370	85.2	2885	84.7
Mixed or other	205	6.5	224	5.7	228	5.3	319	5.5
Black	–	–	–	–	593	6.5	724	6.0
Asian	–	–	–	–	177	3.0	261	3.7
*Missing*	*78*	*2.1*	*79*	*1.7*	*58*	*1.8*	*92*	*2.1*

NVQ = National Vocational Qualifications. FT = Full-time. PT = Part-time. WI% represents proportions after weighting and multiple imputation. The proportions of missing cases reported across study variables (*Missing* rows) are not weighted.

The proportion of young adults who completed post-secondary education (NVQ 3–5) did not increase among men but increased by 9 percentage points (19%) among women. Differences in economic activity at ages 25–26 varied by sex, as the proportion of young adults in: 1) full-time employment decreased from 83% to 75% in men and 63%–58% in women; 2) part-time employment increased from 2% to 7% in men and 13% to 17% in women; 3) unemployment increased from 8% to 9% in men and 3% to 6% in women, and 4) full-time student status increased from 3% to 4% in men and 2% to 5% in women. The proportion of young adults who left their parents to be renters increased from 27% to 34% in men and 30% to 44% in women, whereas home ownership decreased from 36% to 15% in men and 45% to 19% in women between cohorts. In line with trends in conception rates, the proportion of young adults living with children did not significantly change between cohorts (ONS, 2020).

### 3.2 Smoking outcomes across transition milestones at ages 25-26

The remaining tables present the relative risk ratios (RRR) of occasional (Table 2, Table 3) and daily (Table 4, Table 5) smoking across transition milestones among men and women in 1996 and 2015–16.

**Table 2:** Relative risk ratios (RRR) of **occasional smoking among men** across milestones at ages 25–26. 1970 British Cohort study (1996) and Next Steps study (2015–16).

	Men in 1970 cohort *N* = 3764				Men in Next Steps cohort *N* = 3426			
	Partial		Full		Partial		Full	
	RRR	95%CI	RRR	95%CI	RRR	95%CI	RRR	95%CI
**Education (NVQ)**								
No qualifications (ref.)	–		–		–		–	
NVQ 1	1.43	(0.80–2.57)	1.49	(0.82–2.71)	1.10	(0.59–2.06)	1.04	(0.55–1.96)
NVQ 2	1.00	(0.64–1.56)	1.06	(0.67–1.67)	0.89	(0.50–1.56)	0.86	(0.48–1.55)
NVQ 3	1.28	(0.79–2.09)	1.39	(0.84–2.28)	1.07	(0.59–1.93)	1.01	(0.55–1.86)
NVQ 4	1.06	(0.67–1.68)	1.06	(0.66–1.70)	**0.57**	**(0.32**–**0.99)**	**0.55**	**(0.31**–**0.99)**
NVQ 5	0.82	(0.45–1.49)	0.74	(0.40–1.37)	0.80	(0.43–1.48)	0.76	(0.40–1.44)
**Economic activity**								
FT employed (ref.)	–		–		–		–	
PT employed	1.09	(0.53–2.26)	0.98	(0.47–2.02)	0.80	(0.42–1.51)	0.77	(0.40–1.48)
Unemployed	1.42	(0.90–2.23)	1.24	(0.78–1.97)	1.11	(0.63–1.95)	1.02	(0.58–1.80)
FT student	1.07	(0.62–1.84)	0.94	(0.54–1.65)	0.88	(0.48–1.61)	0.91	(0.49–1.69)
Other	0.82	(0.39–1.73)	0.72	(0.34–1.54)	0.79	(0.28–2.19)	0.68	(0.24–1.92)
**Independent living**								
With parents rent-free (ref.)	–		–		–		–	
W/out parents, owning	**0.74**	**(0.55**–**0.99)**	0.92	(0.63–1.33)	0.81	(0.52–1.25)	0.96	(0.60–1.53)
W/out parents, renting	1.27	(0.94–1.72)	**1.49**	**(1.07**–**2.09)**	0.95	(0.67–1.36)	1.06	(0.72–1.56)
Other	1.11	(0.74–1.66)	1.21	(0.81–1.82)	0.97	(0.67–1.41)	0.96	(0.66–1.39)
**Relationship status**								
Single (ref.)	–		–		–		–	
In couple	0.84	(0.64–1.12)	0.83	(0.59–1.16)	0.74	(0.53–1.03)	0.70	(0.48–1.01)
Married	**0.62**	**(0.46**–**0.83)**	**0.65**	**(0.43**–**0.97)**	0.83	(0.48–1.45)	0.76	(0.42–1.38)
Div/Sep/Wid.	0.86	(0.34–2.16)	0.83	(0.32–2.18)	0.10	(0.01–1.27)	0.10	(0.01–1.28)
**Living with children**								
No children (ref.)	–		–		–		–	
One child	1.00	(0.68–1.46)	1.26	(0.81–1.94)	1.08	(0.64–1.82)	1.08	(0.69–2.01)
2+ children	0.57	(0.28–1.16)	0.70	(0.33–1.45)	1.07	(0.58–2.00)	1.07	(0.61–2.30)

Bolded estimates are significant at the p < .05 level. RRR = Relative risk ratio. CI = Confidence interval. NVQ = National Vocational Qualification. FT = Full-time. PT = Part-time. Control variables in partial and full models included: parents' education, parents' housing tenure, mother's age at birth, adolescent health, ethnic group, living in England at ages 25–26, and regular smoking by age 16. Partial and full models included the five milestone variables separately and together, respectively.

**Table 3:** Relative risk ratios (RRR) of **occasional smoking among women** across milestones at ages 25–26. 1970 British Cohort study (1996) and Next Steps study (2015–16).

	Women in 1970 cohort *N* = 4568				Women in Next Steps cohort *N* = 4281			
	Partial		Full		Partial		Full	
	RRR	95%CI	RRR	95%CI	RRR	95%CI	RRR	95%CI
**Education (NVQ)**								
No qualifications (ref.)	–		–		–		–	
NVQ 1	0.70	(0.40–1.19)	0.74	(0.43–1.28)	0.76	(0.37–1.55)	0.71	(0.34–1.47)
NVQ 2	0.98	(0.67–1.43)	1.10	(0.74–1.63)	1.01	(0.55–1.85)	1.04	(0.57–1.92)
NVQ 3	0.79	(0.50–1.27)	0.89	(0.55–1.44)	0.93	(0.51–1.68)	0.92	(0.50–1.71)
NVQ 4	1.00	(0.68–1.48)	0.94	(0.62–1.41)	0.82	(0.45–1.51)	0.85	(0.45–1.60)
NVQ 5	1.14	(0.67–1.95)	0.88	(0.51–1.54)	0.62	(0.33–1.18)	0.59	(0.30–1.16)
**Economic activity**								
FT employed (ref.)	–		–		–		–	
PT employed	1.00	(0.72–1.38)	1.10	(0.75–1.60)	0.89	(0.61–1.30)	0.91	(0.61–1.36)
Unemployed	0.67	(0.28–1.57)	0.47	(0.20–1.12)	0.98	(0.56–1.70)	0.88	(0.49–1.58)
FT student	1.48	(0.80–2.76)	1.01	(0.53–1.91)	0.52	(0.27–1.00)	**0.49**	**(0.24**–**0.98)**
Other	1.00	(0.74–1.35)	0.97	(0.65–1.44)	0.82	(0.50–1.35)	0.86	(0.46–1.59)
**Independent living**								
With parents rent-free (ref.)	–		–		–		–	
W/out parents, owning	**0.63**	**(0.47**–**0.85)**	1.15	(0.81–1.65)	0.80	(0.51–1.26)	1.22	(0.74–2.02)
W/out parents, renting	**1.58**	**(1.16**–**2.14)**	**2.14**	**(1.53**–**2.98)**	**1.50**	**(1.04**–**2.17)**	**1.95**	**(1.33**–**2.86)**
Other	0.76	(0.48–1.21)	1.00	(0.62–1.60)	1.41	(0.92–2.17)	1.51	(0.97–2.35)
**Relationship status**								
Single (ref.)	–		–		–		–	
In couple	0.82	(0.63–1.07)	**0.74**	**(0.55**–**0.99)**	0.80	(0.60–1.07)	**0.73**	**(0.54**–**1.00)**
Married	**0.34**	**(0.26**–**0.44)**	**0.32**	**(0.23**–**0.45)**	**0.30**	**(0.18**–**0.51)**	**0.29**	**(0.16**–**0.51)**
Div/Sep/Wid.	0.71	(0.41–1.25)	0.62	(0.35–1.10)	2.64	(0.82–8.50)	2.61	(0.80–8.47)
**Living with children**								
No children (ref.)	–		–		–		–	
One child	0.88	(0.65–1.19)	1.04	(0.70–1.54)	0.89	(0.60–1.31)	0.92	(0.59–1.43)
2+ children	0.87	(0.61–1.23)	1.03	(0.66–1.61)	0.77	(0.48–1.23)	0.85	(0.48–1.52)

Bolded estimates are significant at the p < .05 level. RRR = Relative risk ratio. CI = Confidence interval. NVQ = National Vocational Qualification. FT = Full-time. PT = Part-time. Control variables in partial and full models included: parents' education, parents' housing tenure, mother's age at birth, adolescent health, ethnic group, living in England at ages 25–26, and regular smoking by age 16. Partial and full models included the five milestone variables separately and together, respectively.

**Table 4:** Relative risk ratios (RRR) of **daily smoking among men** across milestones at ages 25–26. 1970 British Cohort study (1996) and Next Steps study (2015–16).

	Men in 1970 cohort *N* = 3764				Men in Next Steps cohort *N* = 3426			
	Partial		Full		Partial		Full	
	RRR	95%CI	RRR	95%CI	RRR	95%CI	RRR	95%CI
**Education (NVQ)**								
No qualifications (ref.)	–		–		–		–	
NVQ 1	0.83	(0.52–1.33)	0.91	(0.56–1.48)	1.25	(0.75–2.08)	1.23	(0.73–2.08)
NVQ 2	0.75	(0.53–1.05)	0.85	(0.60–1.20)	0.98	(0.61–1.58)	1.16	(0.70–1.93)
NVQ 3	0.80	(0.56–1.15)	0.95	(0.65–1.37)	0.94	(0.56–1.59)	1.07	(0.61–1.86)
NVQ 4	**0.50**	**(0.36**–**0.71)**	**0.54**	**(0.38**–**0.78)**	**0.39**	**(0.23**–**0.67)**	**0.46**	**(0.25**–**0.82)**
NVQ 5	**0.26**	**(0.14**–**0.48)**	**0.24**	**(0.13**–**0.45)**	**0.53**	**(0.29**–**0.98)**	0.61	(0.32–1.18)
**Economic activity**								
FT employed (ref.)	–		–		–		–	
PT employed	1.57	(0.80–3.05)	1.28	(0.65–2.52)	1.38	(0.83–2.30)	1.27	(0.76–2.12)
Unemployed	**1.94**	**(1.34**–**2.83)**	**1.48**	**(1.01**–**2.19)**	**2.09**	**(1.34**–**3.24)**	**1.82**	**(1.18**–**2.82)**
FT student	0.86	(0.48–1.54)	0.85	(0.45–1.60)	0.55	(0.23–1.32)	0.62	(0.26–1.48)
Other	**1.72**	**(1.03**–**2.88)**	1.26	(0.74–2.15)	1.14	(0.61–2.15)	0.93	(0.49–1.76)
**Independent living**								
With parents rent-free (ref.)	–		–		–		–	
W/out parents, owning	**0.64**	**(0.49**–**0.83)**	0.79	(0.56–1.10)	**0.42**	**(0.25**–**0.70)**	**0.58**	**(0.34**–**0.99)**
W/out parents, renting	**1.62**	**(1.23**–**2.13)**	**1.80**	**(1.32**–**2.45)**	1.03	(0.73–1.46)	1.28	(0.87–1.88)
Other	1.28	(0.91–1.79)	1.35	(0.95–1.91)	0.96	(0.66–1.40)	0.98	(0.67–1.43)
**Relationship status**								
Single (ref.)	–		–		–		–	
In couple	1.02	(0.80–1.30)	1.02	(0.76–1.37)	**0.67**	**(0.47**–**0.94)**	**0.56**	**(0.37**–**0.86)**
Married	**0.56**	**(0.44**–**0.73)**	**0.59**	**(0.41**–**0.84)**	**0.47**	**(0.28**–**0.77)**	**0.39**	**(0.22**–**0.69)**
Div/Sep/Wid.	1.38	(0.77–2.47)	1.33	(0.71–2.49)	0.15	(0.02–1.37)	0.18	(0.02–1.54)
**Living with children**								
No children (ref.)	–		–		–		–	
One child	0.97	(0.72–1.31)	1.17	(0.84–1.63)	1.35	(0.83–2.20)	**1.80**	**(1.05**–**3.08)**
2+ children	1.07	(0.69–1.65)	1.21	(0.73–2.02)	1.30	(0.74–2.28)	1.61	(0.90–2.88)

Bolded estimates are significant at the p < .05 level. RRR = Relative risk ratio. CI = Confidence interval. NVQ = National Vocational Qualification. FT = Full-time. PT = Part-time. Control variables in partial and full models included: parents' education, parents' housing tenure, mother's age at birth, adolescent health, ethnic group, living in England at ages 25–26, and regular smoking by age 16. Partial and full models included the five milestone variables separately and together, respectively.

**Table 5:** Relative risk ratios (RRR) of **daily smoking among women** across milestones at ages 25–26. 1970 British Cohort study (1996) and Next Steps study (2015–16).

	Women in 1970 cohort *N* = 4568				Women in Next Steps cohort *N* = 4281			
	Partial		Full		Partial		Full	
	RRR	95%CI	RRR	95%CI	RRR	95%CI	RRR	95%CI
**Education (NVQ)**								
No qualifications (ref.)	–		–		–		–	
NVQ 1	**0.69**	**(0.48**–**0.99)**	0.76	(0.53–1.11)	1.03	(0.60–1.76)	1.01	(0.58–1.76)
NVQ 2	**0.64**	**(0.48**–**0.86)**	0.74	(0.55–1.01)	0.84	(0.52–1.36)	0.97	(0.59–1.60)
NVQ 3	**0.61**	**(0.43**–**0.88)**	0.72	(0.49–1.04)	0.61	(0.35–1.06)	0.72	(0.40–1.27)
NVQ 4	**0.49**	**(0.36**–**0.66)**	**0.49**	**(0.35**–**0.68)**	**0.46**	**(0.28**–**0.77)**	**0.55**	**(0.32**–**0.95)**
NVQ 5	**0.33**	**(0.19**–**0.58)**	**0.27**	**(0.15**–**0.47)**	**0.38**	**(0.21**–**0.71)**	**0.42**	**(0.22**–**0.82)**
**Economic activity**								
FT employed (ref.)	–		–		–		–	
PT employed	0.98	(0.75–1.29)	0.87	(0.63–1.21)	1.26	(0.89–1.77)	1.03	(0.70–1.52)
Unemployed	1.39	(0.85–2.27)	0.90	(0.54–1.47)	**1.96**	**(1.22**–**3.13)**	1.32	(0.80–2.18)
FT student	0.88	(0.46–1.68)	0.71	(0.35–1.43)	1.16	(0.64–2.11)	1.01	(0.50–2.03)
Other	1.21	(0.96–1.53)	0.86	(0.62–1.19)	1.42	(0.95–2.12)	1.10	(0.68–1.76)
**Independent living**								
With parents rent-free (ref.)	–		–		–		–	
W/out parents, owning	**0.64**	**(0.50**–**0.82)**	1.13	(0.82–1.56)	**0.41**	**(0.25**–**0.67)**	0.66	(0.39–1.13)
W/out parents, renting	**1.88**	**(1.46**–**2.43)**	**2.59**	**(1.92**–**3.51)**	1.30	(0.91–1.84)	**1.49**	**(1.02**–**2.18)**
Other	0.94	(0.64–1.38)	1.26	(0.85–1.89)	0.93	(0.61–1.43)	1.26	(0.60–1.43)
**Relationship status**								
Single (ref.)	–		–		–		–	
In couple	0.80	(0.64–1.00)	**0.69**	**(0.53**–**0.90)**	**0.62**	**(0.46**–**0.83)**	**0.62**	**(0.45**–**0.87)**
Married	**0.43**	**(0.34**–**0.53)**	**0.39**	**(0.30**–**0.52)**	**0.37**	**(0.23**–**0.58)**	**0.36**	**(0.22**–**0.59)**
Div/Sep/Wid.	1.09	(0.72–1.65)	0.87	(0.56–1.36)	2.32	(0.89–6.01)	1.86	(0.72–4.83)
**Living with children**								
No children (ref.)	–		–		–		–	
One child	1.00	(0.79–1.28)	1.05	(0.77–1.43)	1.20	(0.85–1.69)	1.04	(0.69–1.57)
2+ children	1.22	(0.94–1.57)	1.20	(0.84–1.72)	1.25	(0.85–1.85)	1.10	(0.68–1.78)

Bolded estimates are significant at the p < .05 level. RRR = Relative risk ratio. CI = Confidence interval. NVQ = National Vocational Qualification. FT = Full-time. PT = Part-time. Control variables in partial and full models included: parents' education, parents' housing tenure, mother's age at birth, adolescent health, ethnic group, living in England at ages 25–26, and regular smoking by age 16. Partial and full models included the five milestone variables separately and together, respectively.

#### 3.2.1 Occasional smoking

Among men aged 26 in the 1970 cohort, two characteristics were associated with occasional smoking: 1) those renting independently had a higher risk of smoking occasionally compared to those living with parents rent-free (RRR = 1.49, 95%CI 1.07–2.09), and 2) those married had a lower risk of smoking occasionally compared to those single (RRR = 0.65, 95%CI 0.43–0.97). Among men aged 25–26 in the Next Steps cohort, one characteristic was associated with occasional smoking: those with further education (NVQ 4) had a lower risk of smoking occasionally compared to those with no qualifications (RRR = 0.55, 95%CI 0.31–0.99).

Among women aged 26 in the 1970 cohort, two characteristics were associated with occasional smoking: 1) those renting independently had a higher risk of smoking occasionally compared to those living with parents rent-free (RRR = 2.14, 95%CI 1.53–2.98), and 2) those partnered and married had a lower risk of smoking occasionally compared to those single (RRR partnered = 0.74, 95%CI 0.55–0.99; RRR married = 0.32, 95%CI 0.23–0.45). Among women aged 25–26 in the Next Steps cohort, three characteristics were associated with occasional smoking: 1) those in full-time studies had a lower risk of smoking occasionally compared to those in full-time employment (RRR = 0.49, 95%CI 0.24–0.98), 2) those renting independently had a higher risk of smoking occasionally compared to those living with parents rent-free (RRR = 1.95, 95%CI 1.33–2.86), 3) those partnered and married had a lower risk of smoking occasionally compared to those single (RRR partnered = 0.73, 95%CI 0.54–1.00; RRR married = 0.29, 95%CI 0.16–0.51).

There were no significant differences in associations among men and women between cohorts based on SUEST results.

#### 3.2.2 Daily smoking

Among men aged 26 in the 1970 cohort, four characteristics were associated with daily smoking: 1) those with further education (NVQ4) or higher education (NVQ 5) had a lower risk of smoking daily compared to those with no qualifications (RRR NVQ4 = 0.54, 95%CI 0.38–0.78; RRR NVQ5 = 0.24, 95%CI 0.13–0.45), 2) unemployment was associated with a higher risk of smoking daily compared to full-time employment (RRR = 1.48, 95%CI 1.01–2.19), 3) independently renting was associated with a higher risk of smoking daily compared to living with parents rent-free (RRR = 1.80, 95%CI 1.32–2.45), and 4) being married was associated with a lower risk of smoking daily compared to being single (RRR = 0.59, 95%CI 0.41–0.84). Among men aged 25–26 in the Next Steps cohort, the five transition variables were associated with daily smoking: 1) those with further education (NVQ 4) had a lower risk of smoking daily compared to those with no qualifications (RRR = 0.46, 95%CI 0.25–0.82), 2) unemployment was associated with a higher risk of smoking daily compared to full-time employment (RRR = 1.82, 95%CI 1.18–2.82), 3) home ownership was associated with a lower risk of smoking daily compared to those living with parents rent-free (RRR = 0.58, 95%CI 0.34–0.99), 4) being partnered and married were associated with a lower risk of smoking daily compared to being single (RRR partnered = 0.56, 95%CI 0.37–0.86; RRR married = 0.39, 95%CI 0.22–0.69), and 5) having one child was associated with a higher risk of smoking daily compared to having no children (RRR = 1.80, 95%CI 1.05–3.08).

We found two significant cohort differences among men based on SUEST results: 1) the association of higher education (NVQ 5) with daily smoking was greater in the 1970 cohort (RRR = 0.24, 95%CI 0.13–0.45) than in the Next Steps cohort (RRR = 0.61, 95%CI 0.32–1.18) (*p* = .044); 2) the association of being partnered with daily smoking was not significant in the 1970 cohort (RRR = 1.02, 95%CI 0.76–1.37) but relatively strong in the Next Steps cohort (RRR = 0.56, 95%CI 0.37–0.86) (*p* = .021).

Among women aged 26 in the 1970 cohort, three characteristics were associated with daily smoking: 1) further education (NVQ4) and higher education (NVQ 5) were associated with a lower risk of smoking daily compared to no qualifications (RRR NVQ4 = 0.49, 95%CI 0.35–0.68; RRR NVQ5 = 0.27, 95%CI 0.15–0.47), 2) independently renting was associated with a higher risk of smoking daily compared to those living with parents rent-free (RRR = 2.59, 95%CI 1.92–3.51), and 3) being partnered and married were associated with a lower risk of smoking daily compared to being single (RRR partnered = 0.69, 95%CI 0.53–0.90; RRR married = 0.39, 95%CI 0.30–0.52). Among women aged 25–26 in the Next Steps cohort, the same characteristics were associated with daily smoking: 1) further education (NVQ4) and higher education (NVQ 5) (RRR NVQ4 = 0.55, 95%CI 0.32–0.95; RRR NVQ5 = 0.42, 95%CI 0.22–0.82), 2) independently renting (RRR = 1.49, 95%CI 1.02–2.18), and 3) being partnered and married (RRR partnered = 0.62, 95%CI 0.45–0.87; RRR married = 0.36, 95%CI 0.22–0.59).

We found one significant cohort difference among women based on SUEST results: the association of independently renting with daily smoking was greater in the 1970 cohort (RRR = 2.59, 95%CI 1.92–3.51) than in the Next Steps cohort (RRR = 1.49, 1.02–2.18) (*p* = .022).

#### 3.2.3 Sensitivity analysis with the four-category outcome

Testing associations in models using the four-category definition of smoking status (never, former, occasional, and daily smoking), we found that never smoking and former smoking at ages 25–26 significantly differed across transition variables. Among men, former smoking (compared to never smoking) in the 1970 cohort was more prevalent among those who were independent owners (RRR = 1.52, 95%CI 1.09–2.13) or renters (RRR = 1.43, 1.02–2.01); in the Next Steps cohort, former smoking was less prevalent among those who had completed higher education (RRR = 0.50, 0.27–0.95). Among women, former smoking (compared to never smoking) in the 1970 cohort was less prevalent among those who completed higher education (RRR = 0.59, 95%CI 0.37–0.97) and more prevalent among full-time students (RRR = 1.94, 95%CI 1.11–3.40), independent renters (RRR = 1.57, 95%CI 1.13–2.18), those in couple (RRR = 1.72, 95%CI 1.30–2.27), and those living with one (RRR = 1.62, 95%CI 1.22–2.15) or multiple children (RRR = 1.48, 95%CI 1.04–2.11). Associations were similar in the Next Steps cohort except for the ones with “full-time student” (RRR = 0.99, 95%CI 0.61–1.60) and “in couple” (RRR = 1.17, 95%CI 0.90–1.53), which were no longer significant.

Findings were similar between the sensitivity analyses and the main analyses presented here (i.e., using “never smoking” instead of “not currently smoking” as the reference category with the additional adjustment for smoking history by age 16). We note however meaningful differences regarding the association of education and parenthood with daily smoking: since former smokers were more likely to be less educated and live with children compared with never smokers, these two variables were associated to a higher degree with daily smoking when compared to never smoking than when compared with not currently smoking (controlling for smoking history).

## 4 Discussion

This study is one of the few attempts to explore how social change in recent decades have influenced the associations between transition milestones and smoking in young adulthood. Comparing the attainment of transition milestones by ages 25–26 across datasets corroborated the fact that, for the later born cohort, the third decade of life has been shaped by longer periods in education, delays in independent living and partnership, and drops in marriage and home ownership. Exploring how these differences related to smoking among men and women at this age, we found that educational attainment, economic activity, independent living, relationship status, and parenthood were each associated with smoking beyond the predisposing roles of social origin and early smoking uptake in 1996 and 2015–16. Partially contrasting with our hypotheses, we found relatively few differences in the associations of transition milestones with smoking between 1996 and 2015–16.

As identified in previous studies, we found that young adults were less likely to smoke if they had higher qualifications and were employed full-time, partnered, and home owners, with relatively few differences by sex (Gagné et al., 2020; Green et al., 2017). Exceptions included that: 1) higher education was a relatively weaker protective factor against daily smoking among men, particularly once other milestones were added to the model; 2) cohabitation with an unmarried partner emerged as a new protective factor against daily smoking among men; and 3) renting independently (i.e., not living with parents) became less pronounced as a risk factor, particularly among women.

The fully-adjusted association of higher education with daily smoking was lower among men in 2015–16, contrasting with the fact that, in the adult population, inequalities in smoking by occupation or economic activity have been relatively stable over the past two decades (Hiscock et al., 2012b; Office for National Statistics, 2019b). We hypothesized that educational attainment – controlling for covariates and other transition milestones – may be less strongly associated with smoking if completing a degree related to fewer social and economic returns by ages 25–26 in the 2010s compared with the 1990s. It may also be that young men who complete a degree have become over time more likely to delay the uptake of healthy practices such as smoking cessation years after ages 25–26. Supporting this, studies found that age-graded differences in smoking across education categories were relatively small at ages 18, rapidly increased between ages 18 and 25, and were likely to continue increasing afterwards (Gagné et al., 2019b, 2020).

A second explanation may be that the “total” effect of education on smoking may be explained to a larger degree by its positive relationship with work and family life transitions associated with cessation over time (Schoon & Lyons-Amos, 2017). For instance, in our results, whereas adjusting for other transition variables did not reduce the size of the association of having completed higher education with daily smoking in the 1970 cohort, doing so slightly reduced the size of this association in the Next Steps cohort (e.g., from RRR = 0.53 to 0.61 for NVQ 5 in men). Supporting this, another study comparing the combination of transition milestones in the 1970 and Next Steps cohorts found an increased proportion of men and women, entitled “left behind”, who had both few qualifications and a high risk of unemployment and singlehood in 2015–16 compared with 1996 (Gagné et al., 2021).

Finally, a third explanation is that the weaker association of education with smoking across cohorts may be the result of the increasing selection of never-smokers into higher education over time (Maralani, 2013). That is, the impact of completing higher education on reducing the risk of daily smoking at ages 25–26 may decrease over time if the early transition into smoking has an increased role in the probability of avoiding higher education and maintaining smoking during young adulthood. This is partially supported by our sensitivity analysis, which suggested that educational differences in daily smoking were larger when compared with never smoking than when compared with former smoking.

The other cohort differences – cohabitating with an unmarried partner as a new protective factor among men and independently renting as a weaker risk factor among women – are likely linked to the rise in alternative forms of living arrangements and partnership over the past three decades in the UK (Stone et al., 2011; Perelli-Harris & Bernardi, 2015). The new benefit observed for non-married partnership among men supports the idea that entering a long-term relationship outside marriage may match the changes in networks, environments, and norms traditionally associated with marriage (Perelli-Harris & Bernardi, 2015). That this change was only present among men is also aligned with the finding that marriage has been more consistently linked to smoking cessation among men compared to women (Homish & Leonard, 2005). Similarly, elongated periods in education, delays in financial independence, and rising housing prices have made the transition into renting an increasingly common experience (Stone et al., 2011; Fiori et al., 2020). The findings suggest that the changes in networks, environments, and norms associated with independent living, historically associated with home buying in the UK, may extend to the larger proportion of young adults who now start their transition to independence by renting (Fiori et al., 2020; Office for National Statistics, 2019a).

We conclude by noting that men were found to be more likely to smoke daily if they had one child (compared to no child) in 2015–16, but not in 1996. Whereas motherhood is associated with smoking cessation, this association has been found to be weaker among fathers and to further vary across social groups, with underprivileged fathers being unlikely to quit smoking (Blackburn et al., 2005; Bottorff et al., 2006; Bricard et al., 2017; O’Donnell et al., 2019). The poorer economic opportunities experienced by young adults today disproportionally affect young fathers, which may make this milestone less likely to be associated with a lower risk of smoking when experienced precociously in more recent years (Neale & Davies, 2016). There may also be selection (i.e., young fathers being more likely to have been smokers beforehand in 2015–16 compared to 1996) that is not fully captured by our statistical adjustment strategy. This hypothesis was again supported by the sensitivity analysis, which suggested that differences in daily smoking by parenthood were larger when comparing with never smoking than when comparing with former smoking.

### 4.1 Strengths & limitations

This study builds on the qualities of the 1970 British Cohort Study and Next Steps to produce robust evidence on the distribution of smoking among British young adults. The project was not pre-registered and the results should be considered exploratory. We highlight that the analyses did not distinguish the timing of transition milestones as a potential effect modifier of their association with smoking. We also did not consider variation across combinations of transition milestones, e.g., using statistical interactions. Their meaning as social role markers and their relationship with smoking are likely to vary across combinations, and it is possible that interactions have also varied over the past twenty years (Gagné et al., 2020, 2021).

We note five other limitations. First, differences in the ages at which covariates were measured across datasets (ages 0–16 in the 1970 cohort and 13–14 in Next Steps) limit the comparability of our adjustment strategy. Second, transition milestones and smoking were measured at the same time point, precluding us from ruling out reverse causality or unobserved confounding. Since cohorts of people followed at the same ages represent a strong tool to disentangle age effects over other longitudinal designs such as panel studies, others may build on the future waves of the Next Steps study to compare the longitudinal associations between young adult transitions and smoking in the datasets used here. Third, the capacity of multiple imputation to mitigate bias depends on the strength of the imputation model. It also cannot adjust for data that is missing-not-at-random (i.e., smokers skipping smoking-related items). Fourth, the occupation-based social class variables derived in the 1996 and 2015-16 samples differed (i.e., the 1970 cohort dataset includes the Registrar-General Social Class scheme and the Next Steps cohort dataset includes the National Statistics Socioeconomic Classification scheme), precluding us from directly comparing the role of social class over time. Finally, the behavioural characteristics of occasional and daily smokers have changed over time: e.g., daily smokers in 2016 include fewer heavy smokers than in 1996 (Office for National Statistics, 2019b). The full comparison of smoking over time therefore needs a range of other definitions and modelling strategies beyond those tested in this study.

### 4.2 Conclusions

Given the health benefits of early smoking cessation, young adulthood represents a key window of opportunity for tobacco control. Contrasting with older age groups, young adults rapidly experience a range of social role transitions that have marked implications on the resources, networks, environments, and norms shaping health practices. Whereas smoking and the transition to adulthood have considerably changed in recent decades, key transition milestones were associated to a similar degree with occasional and daily smoking at ages 25–26 in 1996 and 2015–16. Planning towards the reduction of social inequalities in smoking at the population level is likely to benefit from considering its progression during young adulthood. Whereas interventions have been found to prevent initiation and promote cessation among young adults (including some tailored to this age group), there remains a lack of evidence on the magnitude of benefits across social groups (Fanshawe et al., 2017; Hill et al., 2014; Kock et al., 2019; Villanti et al., 2020). Prevention efforts could be strengthened by further considering the challenges faced by young adults across work and family transitions, which has been a relatively low priority of intervention research despite the size of differences in risks across groups (Bader et al., 2007).

## Ethical statement

We declare having no conflicts of interest. No funding organisations were involved in the writing of the manuscript or the decision to submit the manuscript. No ethical approval was required for the analysis of the 1970 British Cohort Study and Next Steps datasets.

## Author statement

**TG:** Conceptualization, Methodology, Formal Analysis, Writing – Original Draft, Writing – Review & editing.

**IS:** Methodology, Writing – Review & editing, Supervision.

**AS:** Methodology, Writing – Review & editing, Supervision.
